# A Compound Hop Index for Assessing Soccer Players’ Performance

**DOI:** 10.3390/jcm11010255

**Published:** 2022-01-04

**Authors:** Łukasz Oleksy, Aleksandra Królikowska, Anna Mika, Maciej Kuchciak, Daniel Szymczyk, Marian Rzepko, Grzegorz Bril, Robert Prill, Artur Stolarczyk, Paweł Reichert

**Affiliations:** 1 Orthopaedic and Rehabilitation Department, Medical Faculty, Medical University of Warsaw, 02-091 Warsaw, Poland; loleksy@oleksy-fizjoterapia.pl (Ł.O.); artur.stolarczyk@wum.edu.pl (A.S.); 2 Oleksy Medical & Sports Sciences, 37-100 Łańcut, Poland; 3 Polish Strength and Conditioning Association, 44-141 Gliwice, Poland; 4 Physiotherapy and Sports Centre, Rzeszow University of Technology, 35-959 Rzeszow, Poland; g.bril@prz.edu.pl; 5 Ergonomics and Biomedical Monitoring Laboratory, Department of Physiotherapy, Faculty of Health Sciences, Wroclaw Medical University, 50-367 Wroclaw, Poland; 6 Institute of Clinical Rehabilitation, University of Physical Education in Krakow, 31-571 Krakow, Poland; anna.mika@awf.krakow.pl; 7 Institute of Physical Culture Sciences, Rzeszow University, 35-310 Rzeszow, Poland; mkuchciak@ur.edu.pl (M.K.); mrzepko@ur.edu.pl (M.R.); 8 Institute of Health Sciences, Medical College, Rzeszow University, 35-310 Rzeszow, Poland; dszymczyk@ur.edu.pl; 9 Center of Orthopaedics and Traumatology, University of Brandenburg an der Havel Theodor Fontane, 14770 Brandenburg, Germany; Robert.Prill@mhb-fontane.de; 10 Department of Trauma Surgery, Faculty of Medicine, Wroclaw Medical University, 50-367 Wroclaw, Poland; pawel.reichert@umw.edu.pl

**Keywords:** athletic training, injury prevention, performance measure, soccer, sports medicine

## Abstract

Athletes regularly have to pass a series of tests, among which one of the most frequently used functional performance measures are single-leg hop tests. As the collected individual results of tests constitute a large amount of data, strategies to decrease the amount of data without reducing the number of performed tests are being searched for. Therefore, the study aimed to present an effective method to reduce the hop-test battery data to a single score, namely, the Compound Hop Index (CHI) in the example of a soccer team. A male, first-league soccer team performed a battery of commonly used single-leg hop tests, including single hop and triple hop for distance tests and the six-meter timed hop test. Gathered data, including Limb Symmetry Indexes of the three tests, normalized to body height for the single- and triple-hop-tests distance separately for right and left legs, and the time of the six-meter timed hop test separately for right and left legs were standardized to z-scores. Consecutively, the z-scores were averaged and formed CHI. The developed CHI represents a novel score derived from the average of z-scores that significantly reduces, clarifies, and organizes the hop performance-measures data.

## 1. Introduction

Athletic development and status are often tested with standardized performance-based tests. Test batteries are carried out for different purposes like functional diagnostic, injury prevention, or return-to-sport decision-making. The collected results of individual tests build up a large amount of data [1,2,3]. Some of the most frequently used functional performance measures are single-leg hop tests [1]. In general, the distance-based and time-based hop tests are used to detect functional limb asymmetry with the between-limbs differences expressed by the Limb Symmetry Index (LSI). [4]. Single-leg hop tests are often used and reported in an examination context, for example, in return-to-sport decision-making for patients after anterior cruciate ligament (ACL) reconstruction [4,5,6,7]. Nonetheless, some authors indicate that interpreting the LSI’s value alone is insufficient [8,9,10]. For further improvement of hop-test validity, recent studies recommend normalizing the hop distances obtained to the body height of the examined person [11,12]. Single-leg hop tests are partially criticized for deficient functional performance evaluation of ACL-reconstructed patients [13]. Not all hop tests might equally be sensitive for detecting between-limbs differences in terms of functional performance [4,14]. Significant issues concerning hop tests are to be seen in terms of subquality criteria like the excellent economy and simplicity. Only a few and cheap pieces of equipment are required; therefore, in-field testing can be performed easily. It is also essential that the single-leg hop tests provide some quantifiable measure that can be evaluated at consecutive points in time with broadly accepted parameters like the hop distance, the between-limbs symmetry, and the time of performing the test [4]. It was proven in many settings that single-leg hop tests provide good to excellent reliability levels in healthy individuals [15,16] and ACL-reconstructed patients [7,17,18]. Because the analysis of the results of individual tests can be time-consuming and its variability may cause confusion, some authors propose identifying fewer but more predictive tests to reduce the number of data [19,20]. In contrast, others provide strategies to decrease the number of data without reducing the number of performed tests.

As the literature shows, the development of composite scores is not a new idea in functional diagnostics. Examples are gait indices commonly used to assess overall pathology and outcomes from studies with instrumented gait analyses [21]. The three well-known gait indices are the Gillette Gait Index (GGI) [22], the Gait Deviation Index (GDI) [23], and the Gait Profile Score (GPS) [24]. The gait indices themselves have many advantages and disadvantages [21]. They are also calculated in different ways [22,23,24]. Still, they are all going for the same issue: the reduction in a large-gait analysis dataset to a single number [21,22,23,24]. Even for ACL-reconstructed patients, scores have been used to describe functional status five years postoperatively [25]. When it comes to sports-medicine decision-making, there is a newly developed score by Oleksy et al. (2021), the Composite Score of Readiness (CRS), providing a single score for return to sport after ACL reconstruction [26]. The CRS was developed following the index called the Total Score of Athleticism (TSA), a single score of an athlete’s holistic athleticism, that was introduced by Turner (2014) [2] using hypothetical data from a fitness-testing battery and consecutively used among soccer teammates [27]. When designing the TSA, Turner et al. (2014) used different components of fitness that collectively define an athlete’s athleticism [2]. Turner et al. (2014) wanted to judge how the athletes did holistically, that is, to have some measure of general athleticism, where moderate scores across all tests may be more beneficial to performance than scoring high in some while doing terrible in others [2,3]. All in all, a single score can precisely determine the size of an athlete’s deficits [21]. What’s more, on the example of single-hop tests battery, instead of examining the effects of individual tests, raising one score seems to be a user-friendly solution.

Therefore, the study aimed to present a Compound Hop Index (CHI) represented by a single score of an athlete’s hop performance using data from a hop-testing battery in soccer players.

## 2. Materials and Methods

The observational single time point, non-randomly sampled cohort study was conducted according to the Declaration of Helsinki’s ethics guidelines and principles. The study was approved by the Bioethics Committee at the Medical University of Wroclaw, Wroclaw, Poland (approval number KB-351/2021). All of the study participants were informed of the purpose and approach to be used and signed a written informed-consent form.

### 2.1. Test–Retest Reliability of the Compound Hop Index

Fourteen healthy male recreational athletes, indicated as Group T-R, without known cardiovascular or orthopedic problems were recruited from the physical-education student population of the university to participate in the intra-day and inter-day test–retest reliability of the CHI (age x = 23.43 ± 1.16 years; bodyweight x = 78.93 ± 10.01 kg; body height x = 1.81 ± 0.08 m). All participants in the Group T-R underwent the single-leg hop tests battery three times according to the methodology described below in Section 2.3. The battery of tests was performed twice on the same day and the third time the next day. The rest time between the first battery of tests and the second one exceeded 90 min. The participants were asked to maintain their regular training regimens during the experimental period and not to participate in any vigorous physical activity between the three occasions of batteries of tests being performed. The batteries of tests were carried out at the same time of the day. Consecutively, the CHI for each test session was calculated according to subsection Section 2.4.

### 2.2. Participants

The initial sample of the study included 45 male soccer players from the one Polish first-league soccer team, precisely the first team and the reserve team that included backup players for the first team. The inclusion criteria were: no history of musculoskeletal injuries or being cleared to play by a medical specialist in the case of sustaining injuries in the past. The decision for return to sport was based on the time, clinical examination, patient-reported, and performance-based criteria [28]. Two soccer players were excluded because they had sustained injuries and were not cleared yet to play by a medical specialist. Therefore, the final studied group included 43 male soccer players (age x = 24.31 ± 5.06 years; body weight x = 80.77 ± 7.47 kg; body height x = 1.84 ± 0.06 m). Among included soccer players, three were more than nine months after a primary unilateral arthroscopic anterior cruciate ligament reconstruction using autologous semitendinosus graft. Five players sustained ankle sprains followed by conservative treatment more than nine months previously. Three players reported grade I or “mild” lower limb muscle injury [29], followed by conservative treatment more than six months before research.

### 2.3. Single-Leg Hop Tests Battery

In the studied group of soccer players, the single-leg hop tests battery was performed on one occasion. The battery was routinely carried out as a part of the pre-seasonal assessment of soccer players. Therefore, the players knew the test protocol very well as they were regularly tested. In both groups, namely, Group T-R and the studied group of soccer players, the tests were carried out on a soccer field by one trained and well-experienced examiner. For standardization purposes, participants were asked to abstain from unaccustomed strenuous exercise for at least 24 h before the tests and to avoid eating a heavy breakfast in the morning before the evaluation and within two hours before the tests. The participants were dressed in comfortable sports outfits and soccer cleats. A 10-min long FIFA 11+ soccer-specific warm-up preceded the assessment [30]. To ensure that the participants were familiar and comfortable with the test protocol, they were allowed to practice. Still, the practice trials were limited to three per leg to prevent potential fatigue.

**Figure 1:**
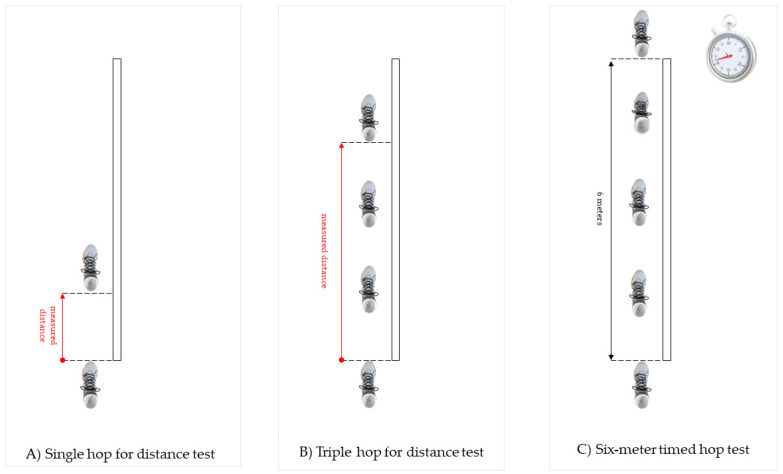
Schematic diagram of the performed battery of three single-leg hop tests, including, consecutively.

Then, the participants performed a battery of three single-leg hop tests, including, consecutively, a single hop for the distance test, a triple hop for the distance test, and the six-meter timed hop test [16,18,31,32,33]. The test order was randomly assigned to each participant. There were 5-min-long rests between particular tests. All of the tests were performed bilaterally, starting with the right leg. In the single hop and triple hop for distance tests, the aim was to hop as far as possible on one leg with a controlled landing. In the six-meter timed hop test, the participant was instructed to jump as fast as possible on a single leg over a distance of six meters. The maximum distance of the two trials of each test was used for analysis. The duration of the timed hop test was measured using a stopwatch. The shortest time of the two trials indicating a better result was used for further analysis. The schematic diagram of the performed battery of the three single-leg hop tests is presented in Figure 1.

### 2.4. Calculation of the Compound Hop Index

The CHI was calculated using Microsoft Office Excel 365 Personal (Microsoft Corporation, Redmond, WA, USA). A detailed description of the calculation of CHI on the hypothetical was presented in Description of the Calculation of Compound Hop Index in Microsoft Office Excel attached as a supplemental file (Supplementary Material S1).

At first, there were calculated z-scores for the given parameters, namely, the normalized single-hop test distance separately for right and left leg, the normalized single-hop test distance LSI, the normalized triple-hop test distance separately for right and left leg, normalized triple-hop test distance LSI, the time of the six-meter timed hop test separately for right and left leg, and the time of the six-meter timed hop test LSI. The formula for calculating a z-score is z = (x − μ)/σ, where x is the raw score, μ is the population mean, and σ is the population standard deviation. As the formula shows, the z-score is simply the raw score minus the population mean divided by the population standard deviation. Computing a z-score was based on an arithmetic mean and the standard deviation of an analyzed parameter of the complete studied group. Consecutively, the arithmetic mean of z-scores of analyzed parameters was computed to form a single score. It is worth noting that in the six-meter timed hop test time, conversely to the remaining tests, a higher value indicates worse performance; the result was multiplied by negative 1.

The z-score, also called a standard score, represents the number of standard deviations by which the value of a raw score obtained by a particular participant is above or below the mean value in a given team of an analyzed parameter. Scores above the team’s mean had positive standard scores, while those below the mean had negative standard scores.

### 2.5. Statistical Analysis

TIBCO Statistica™ Version 13.3 (TIBCO Software Inc., Palo Alto, CA, USA) and Microsoft Office Excel 365 Personal (Microsoft Corporation, Redmond, WA, USA) were used for the statistical analysis.

The collected parameters during single-leg hop tests battery were: the single-hop test distance separately for right and left leg (m), the triple-hop test distance separately for right and left leg (m), and the time of the six-meter timed hop test separately for right and left leg (s). The single-hop and the triple-hop distance values were divided by the body height (m) and expressed as a normalized single-hop test distance and triple-hop test distance separately for the right and left leg (m*m^−1^). Consecutively, the side-to-side differences were expressed as the Limb Symmetry Index (LSI) for particular hop tests, with 100 representing complete symmetry between limbs. The LSI was calculated as a lower value/higher value × 100% [34]. For the distance-based tests, the better score was the higher value (larger distance). The better score was a shorter time (lower value) for the time-based test.

Out of this data pool, the normalized single-hop test distance separately for right and left leg, the normalized single-hop test distance LSI, the normalized triple-hop test distance separately for right and left leg, the normalized triple-hop test distance LSI, the time of the six-meter timed hop test separately for right and left leg, and the time of the six-meter timed hop test LSI were statistically analyzed.

The arithmetic mean (x) and the standard deviation (SD) were calculated beside for the above-mentioned parameters and participants’ age, body mass, and height. The analyzed parameters were normally distributed according to a performed Shapiro–Wilk test.

In the Group T-R, intraclass correlation coefficients (ICC; Shrout and Fleiss model 2) were calculated to analyze the test–retest results. The guidelines described by Cicchetti and Sparrow were used to assess reliability coefficients; namely, 0.40 was considered poor, 0.40–0.59 was considered fair, 0.60–0.74 was considered good, and 0.75 and more was considered excellent.

In the studied group of soccer players, the linear Pearson’s correlation coefficient (*r*) was calculated to assess any relationship between (1) LSIs of the three performed tests; (2) the single-hop test distance LSI and the normalized single-hop test distance separately for right and left leg; (3) the triple-hop test distance LSI and the normalized single-hop test distance separately for right and left leg; (4) the six-meter timed-hop test LSI and the time of the six-meter timed hop test separately for right and left leg. The magnitudes of the bivariate associations were classified as negligible (0.00–0.30), low (0.31–0.50), moderate (0.51–0.70), high (0.71–0.90), and very high (0.91–1.00) [35]. Additionally, the coefficient of determination, the *r*-squared (*r*^2^), was calculated to give a proportion of variance (fluctuation) of one variable predictable from the other variable. The *r*^2^ represents the percentage of data points closest to the line of best fit. The statistical significance was set at *p* < 0.050.

## 3. Results

The intra-day reliability of the CHI was found to be on an excellent level (ICC = 0.98). Even though the ICC for the inter-day CHI comparison was lower than for the intra-day reliability, it was still on an excellent level (ICC = 0.97).

The descriptive statistics, including the arithmetic mean and the standard deviation for the normalized single-hop test distance, the normalized triple-hop test distance, and the time of the six-meter timed hop test in the studied group of soccer players, are presented in Table 1.

There was no correlation between the single-hop test LSI and the six-meter timed hop test LSI (*r* = 0.299, *p* = 0.052). There was also no correlation between the triple-hop test LSI and the six-meter timed hop test LSI (*r* = 0.181, *p* = 0.246). Even though the single-hop test LSI was statistically significantly positively associated with triple-hop test LSI (*r* = 0.320, *p* = 0.036), the association level was low. Only 10% of the total variation in the single-hop test LSI can be explained by the positive linear relationship between the single-hop test LSI and the triple-hop test LSI (*r^2^* = 0.102).

LSI’s single-hop test distance was statistically significantly positively correlated with the right-leg normalized distance as presented in Table 2; however, the association level was low (*p* = 0.029; *r* = 0.333). Only 11% of the total variation in the single-hop test LSI can be explained by the positive linear relationship between the single-hop test LSI and the right-leg normalized distance (*r^2^* = 0.111). No correlations were noted between the single-hop test distance LSI and the left-leg normalized distance. There were no correlations between the triple-hop test distance LSI and the normalized single-hop test distance separately for the right and left leg. No correlation between the six-meter timed hop test LSI and the right leg time was indicated. There was determined a statistically significant negative correlation (*p* ≤ 0.001; *r* = −0.528) between the six-meter timed hop test LSI. It should be indicated that the association was on a moderate level. Only 28% of the total variation in the six-meter timed hop test LSI can be explained by the negative linear relationship between the LSI and left leg time (*r*^2^ = 0.279).

**Figure 2:**
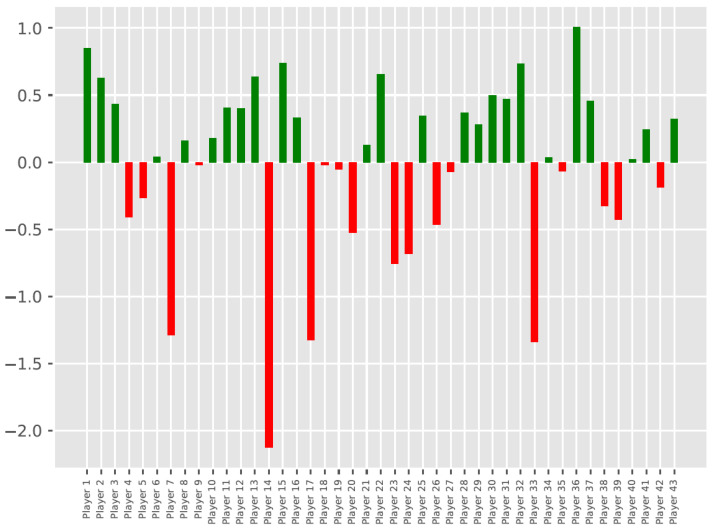
Visualization of the comparative analysis of CHI.b obtained among players in the team with CHI.a color-coded according to the RAG rating. Zero represents the team mean in terms of hop performance. Bars above the zero line represent athletes better than mean, while bars below zero indicate worse-than-mean athletes.

The results in the form of ranking of athletes among the studied group were shown on a bar chart (Figure 2). Additionally, the calculated CHI for particular players was presented in a Supplementary file S2. The arithmetic mean provides the average score of a measured parameter, while the standard deviation provides the dispersion of data from the arithmetic mean. For a z-score, all data are converted to have a mean of zero and a standard deviation of one. So, for example, the CHI for Player 36 exceeding 1.20 indicated that the player scored above the team’s mean by 1.20 of the standard deviation.

Analogously to TSA [2], the CHI scores were color-coded to represent a RAG rating that refers to red and green colors. It is a system of indicating in red players representing the danger zone, who negatively differ from the average of the entire team in terms of hop performance. Conversely, the players in a green safe zone are better than the team’s average. The height of the bar determines how much the particular players positively or negatively deviate from the team’s average.

## 4. Discussion

The study aimed to present a complete score of an athlete’s hop performance among teammates using real data from a hop testing battery in soccer players, namely, the Compound Hop Index (CHI). The evolved CHI is an average of the z-scores of the LSIs of the single-hop test distance, the triple-hop test distance, and the time of the six-meter timed hop test, as well as the normalized values separately for the right and left leg of the single-hop test distance, the triple-hop test distance, and the six-meter timed hop test time. The CHI reduces the hop performance-measures results and is less likely to overestimate knee function than analysis of LSIs values alone.

Functional testing of lower limbs mainly involves agility runs, vertical jumps, balance tests, and hop tests [31,32,36,37]. Hop testing was first cited at the beginning of the 1980s [38] and consecutively has been commonly used since the 1990s [31,36,39] as a functional measure because it mimics dynamic movements performed during athletic participation [1]. The hop tests are suggested to be the most consistent predictor of subsequent RTS in patients after knee-joint anterior-cruciate-ligament reconstruction [40], and they present good reliability [32]. These tests’ frequent usage is partly due to their practical utility and ease of administration [4,41]. The administration of three or four hop tests as the functional component in RTS assessment is common [5,8,42,43,44]. On the other hand, some authors indicated that including all four hop tests as an assessment battery might not be necessary [41]. In the present study, no relationships were found between all aggregate measures that form CHI, or the found correlations were on a low level. It can be concluded that none of the tests is redundant to each other, further leading to the assumption that all tests need to be performed and to be separately included for composing CHI as an expression of hop performance with a significantly reduced number of presented data.

Additionally, because of normalizing performance in regard to an athlete’s body height, CHI is less likely to overestimate the hop performance than the usage of LSI. It should be of high interest because some authors have highlighted that caution should be warranted for LSI use in side-to-side comparison [8,45,46] for two main reasons. Firstly, with the LSI value 100 determining exact inter-limbs symmetry, the minimal symmetry benchmark being acceptable persists as debatable [1]. There have been reports that the LSI in healthy individuals varies with sex, weekly physical activity, and body mass index [1], indicating that its minimum acceptable value is variable. In the RTS decision-making previously, the minimal permissible LSI exceeded 80 to 85 [31,47,48,49]. More recent studies reported LSI in healthy individuals to amount from 84 to 96 [50], but there are also authors reporting 90–96 or even 100 in some hop tests [1]. Potential higher influence is given, especially in RTS assessment, through leg dominance remaining an essential issue in minimal acceptable LSI determination [2,51]. In healthy individuals, the dominant limb may perform even 5% better than the non-dominant one [1]. All potential LSI predictor variables are still not known. Secondly, the LSI masks a bilateral poor functional performance that may overestimate the interpretation of test results [1,2,19,27]. Therefore, it is recommended to normalize the hop distances obtained in the single leg tests to the examined person’s body height [11,12]. Additionally, the percentage relation of the single-leg hop distance in the involved limb to the body height has been determined to be associated with return-to-sport status in ACL-reconstructed patients [11].

Apart from the LSI, when setting test thresholds, many features and aspects considered to be included, like the type and level of sport, players’ age, maturation, or sex [52,53], and the established norms for hop performance available in the literature [1,54] are not always relevant for a given team. One of the CHI advantages is that it ranks an athlete among other teammates setting benchmarks and training goals that are realistic to achieve. For now, the CHI will not be relevant for the RTS decision-making compared to other patients attending physiotherapy. Still, it can be used to check a player after an injury against the rest of the team in terms of cumulative hop performance. Calculating CHI for a particular athlete or patient is possible once we have available normative arithmetic mean and standard deviation in the literature that we can refer to.

The CHI calculation can be performed step by step using a spreadsheet in Microsoft Excel for Windows, macOS, Android, and iOS; therefore, it is commonly available. By visualizing and color-coding according to the RAG rating CHI on a bar chart, sports professionals and athletes get graphical feedback, indicating how bad or how well each athlete did on hop performance tests relative to teammates [2,3]. It may also help identify the team players with unknown conditions that might place them at risk from sports performance even if they do not present any signs or symptoms. The CHI itself will not identify the problem affecting the performance but will indicate that it exists.

CHI is a strategy to reduce data from the hop tests being a part of a battery of assessments, so it should never be considered a standalone evaluation for complex performance issues. Even though the single-leg hop-test results are associated with muscular strength, endurance, neuromuscular coordination, and joint stability [55,56], they did not provide clinicians with enough information to make evidence-based decisions concerning isolated muscle group strength [57]. Additionally, it should be remembered that physical performance is only one of the elements of an athlete’s readiness to sport [58,59,60], and it does not capture neurocognitive deficits or neurophysiologic dysfunction [61].

The study presented an effective method for reducing the hop-test battery data to a single score. Still, some limitations need to be addressed. This CHI is able to synthesize the mass of hop-test data, but the interpretation in terms of content validity stays the same as with hop tests. Therefore, it is still not possible to clarify the predictive value for injuries or the value of CHI for sporting performance, which stays the same as with the single-hop tests or the LSI being regularly used with actually few possible causal conclusions. Showing that the included tests are not redundant to each other addresses the necessity of the individual included tests without finally clarifying the value of each one. In addition to this hop test index, further research on the value of the single tests for a test battery in the individual context must be addressed. In this term, it must be mentioned that this study was performed in only one particular setting. Reliability issues always belong to the included population and the setting they are evaluated in. Therefore, transferring results to other settings should be done with caution.

## 5. Conclusions

The present study introduced a novel approach for analyzing hop performance in a given team represented by a single score derived from the average of z-scores, namely, the Compound Hop Index (CHI). The developed CHI is a tool that reduces and organizes data of hop-test measures. It represents a specific trend towards the user-friendly analysis of data results among teammates.

## Figures and Tables

**Table 1:** Summary of the results obtained from the performed battery of tests.

Analyzed Parameter	Studied Leg	x	SD
Normalized single-hop test distance (m*m^−1^)	Right	1.18	0.10
	Left	1.19	0.11
	LSI	96.46	3.53
Normalized triple-hop test distance (m*m^−1^)	Right	3.80	0.26
	Left	3.82	0.31
	LSI	95.56	3.50
Time of the six-meter timed hop test (s)	Right	1.41	0.11
	Left	1.41	0.13
	LSI	95.03	5.10

The values are expressed as arithmetic mean (x) and standard deviation (SD). The single-hop and triple-hop distance raw values were normalized to the player’s body height. LSI, Limb Symmetry Index.

**Table 2:** Correlations between single-hop test distance LSI and * normalized single-hop test distance separately for right and left leg; triple-hop test distance LSI and * normalized single-hop test distance separately for right and left leg, and six-meter timed hop test LSI and ** time of the six-meter timed hop test separately for right and left leg.

Analyzed Test	Right-Leg Normalized Distance * or Time **	Left-Leg Normalized Distance * or Time **
Single-hop test distance LSI	*p* = 0.029; *r* = 0.333	*p* = 0.164; *r* = 0.216
Triple-hop test distance LSI	*p* = 0.837; *r* = 0.032	*p* = 0.448; *r* = 0.119
Six-meter timed hop test LSI	*p* = 0.056; *r* = −0.294	*p* ≤ 0.001; *r* = −0.528

The values are expressed as correlation coefficient (*r*) and *p*-values. LSI, Limb Symmetry Index.

## Data Availability

The data generated during the current study are available from the corresponding author on reasonable request.

## Supplementary Materials

The following are available online at https://www.mdpi.com/article/10.3390/jcm11010255/s1: Material S1: Description of the Calculation of Compound Hop Index in Microsoft Office Excel; Supplementary Material file S2: List of calculated CHI for particular players of a given team.
