# Supplementary material for: A Compound Hop Index for Assessing Soccer Players’ Performance
Source: J Clin Med. 2022 Jan 4;11(1):255. doi: 10.3390/jcm11010255 (PMC8745790; doi:10.3390/jcm11010255)
Supplement: Supplementary file 1 [file jcm-11-00255-s001.zip › jcm-1495912-supplementary/S1_CHI Calculation Instruction.pdf]

Description of the Calculation of Compound Hop Index* in Microsoft Office Excel

* The description refers to the CHI.b in the present manuscript.

1. Enter data as presented in Figure S1 with one athlete of a given team per row, with body height expressed in meters (m) and test results in consecutive columns across the top, including single-leg hop test distance separately for left and right leg (m), triple hop test distance separately for left and right leg (m), and six-meter timed hop test time separately for left and right leg expressed in seconds (s).

A12 | fx

	A	B	C	D	E	F	G	H	I
1	Consecutive players	Body height [m]	Single-leg hop test distance left leg [m]	Single-leg hop test distance right leg [m]	Triple hop test distance left leg [m]	Triple hop test distance right leg [m]	Six-meter timed hop test time left leg [s]	Six-meter timed hop test time right leg [s]	
2	Player 1	1.83	2.33	2.30	7.65	7.98	1.34	1.28	
3	Player 2	1.81	2.28	2.32	6.95	7.36	1.31	1.29	
4	Player 3	1.80	2.11	2.13	6.88	6.85	1.34	1.32	
5	Player 4	1.91	2.01	2.06	6.49	7.05	1.35	1.39	
6	Player 5	1.84	1.99	1.95	6.63	6.51	1.44	1.43	
7	Player 6	1.82	2.11	2.10	6.69	6.65	1.40	1.46	
8	Player 7	1.85	2.04	1.83	6.82	6.16	1.48	1.61	
9	Player 8	1.80	2.10	2.13	6.88	7.02	1.45	1.36	
10	Player 9	1.83	2.14	2.01	6.77	7.08	1.34	1.36	
11	Player 10	1.88	2.12	2.20	6.97	7.10	1.34	1.32	
12									

Figure S1 The method of preparing data from individual tests performed on the example of a 10-person team.

2. Normalize the single-leg hop test distance (m) and triple hop test distance (m) separately for the right and left legs by dividing the test result of the particular athlete by their body height (m) as presented in Figure S2 and Figure S3. The normalized values are expressed in m*m^{-1}.

B2 | fx | =C2/B2

	A	B	C	D	E	F	G	H	I	J
1	Consecutive players	Body height [m]	Single-leg hop test distance left leg [m]	Single-leg hop test distance right leg [m]	Triple hop test distance left leg [m]	Triple hop test distance right leg [m]	Six-meter timed hop test time left leg [s]	Six-meter timed hop test time right leg [s]	Normalized single-leg hop test distance left leg [m*m^{-1}]	
2	Player 1	1.83	2.33	2.30	7.65	7.98	1.34	1.28	=C2/B2	
3	Player 2	1.81	2.28	2.32	6.95	7.36	1.31	1.29		
4	Player 3	1.80	2.11	2.13	6.88	6.85	1.34	1.32		
5	Player 4	1.91	2.01	2.06	6.49	7.05	1.35	1.39		
6	Player 5	1.84	1.99	1.95	6.63	6.51	1.44	1.43		
7	Player 6	1.82	2.11	2.10	6.69	6.65	1.40	1.46		
8	Player 7	1.85	2.04	1.83	6.82	6.16	1.48	1.61		
9	Player 8	1.80	2.10	2.13	6.88	7.02	1.45	1.36		
10	Player 9	1.83	2.14	2.01	6.77	7.08	1.34	1.36		
11	Player 10	1.88	2.12	2.20	6.97	7.10	1.34	1.32		
12										

Figure S2 The method of normalizing the single-leg hop test distance result by dividing it by the athlete's body height.

Consecutive players	Body height [m]	Single-leg hop test distance left leg [m]	Single-leg hop test distance right leg [m]	Triple hop test distance left leg [m]	Triple hop test distance right leg [m]	Six-meter timed hop test time left leg [s]	Six-meter timed hop test time right leg [s]	Normalized single-leg hop test distance left leg [m*m^{-1}]	Normalized single-leg hop test distance right leg [m*m^{-1}]	Normalized triple hop test distance left leg [m*m^{-1}]	Normalized triple hop test distance right leg [m*m^{-1}]
Player 1	1.83	2.33	2.30	7.65	7.98	1.34	1.28	1.27	1.26	4.18	4.36
Player 2	1.81	2.28	2.32	6.95	7.36	1.31	1.29	1.26	1.28	3.84	4.07
Player 3	1.80	2.11	2.13	6.88	6.85	1.34	1.32	1.17	1.18	3.82	3.81
Player 4	1.91	2.01	2.06	6.49	7.05	1.35	1.39	1.05	1.08	3.40	3.69
Player 5	1.84	1.99	1.95	6.63	6.51	1.44	1.43	1.08	1.06	3.60	3.54
Player 6	1.82	2.11	2.10	6.69	6.65	1.40	1.46	1.16	1.15	3.68	3.65
Player 7	1.85	2.04	1.83	6.82	6.16	1.48	1.61	1.10	0.99	3.69	3.33
Player 8	1.80	2.10	2.13	6.88	7.02	1.45	1.36	1.17	1.18	3.82	3.90
Player 9	1.83	2.14	2.01	6.77	7.08	1.34	1.36	1.17	1.10	3.70	3.87
Player 10	1.88	2.12	2.20	6.97	7.10	1.34	1.32	1.13	1.17	3.71	3.78

Figure S3 Calculated normalized values for single-leg and triple hop test distances separately for left and right legs.

3. Calculate the Limb Symmetry Index, LSI for single-leg hop test distance, triple hop test distance, and six-meter timed hop test time by dividing the worse score by the better score, multiplied by 100 as presented in Figure S4 and Figure S5.

=(J2/I2)*100

Consecutive players	Body height [m]	Single-leg hop test distance left leg [m]	Single-leg hop test distance right leg [m]	Triple hop test distance left leg [m]	Triple hop test distance right leg [m]	Six-meter timed hop test time left leg [s]	Six-meter timed hop test time right leg [s]	Normalized single-leg hop test distance left leg [m*m^{-1}]	Normalized single-leg hop test distance right leg [m*m^{-1}]	Normalized triple hop test distance left leg [m*m^{-1}]	Normalized triple hop test distance right leg [m*m^{-1}]	Single-leg hop test distance LSI
Player 1	1.83	2.33	2.30	7.65	7.98	1.34	1.28	1.27	1.26	4.18	4.36	=(J2/I2)*100
Player 2	1.81	2.28	2.32	6.95	7.36	1.31	1.29	1.26	1.28	3.84	4.07	
Player 3	1.80	2.11	2.13	6.88	6.85	1.34	1.32	1.17	1.18	3.82	3.81	
Player 4	1.91	2.01	2.06	6.49	7.05	1.35	1.39	1.05	1.08	3.40	3.69	
Player 5	1.84	1.99	1.95	6.63	6.51	1.44	1.43	1.08	1.06	3.60	3.54	
Player 6	1.82	2.11	2.10	6.69	6.65	1.40	1.46	1.16	1.15	3.68	3.65	
Player 7	1.85	2.04	1.83	6.82	6.16	1.48	1.61	1.10	0.99	3.69	3.33	
Player 8	1.80	2.10	2.13	6.88	7.02	1.45	1.36	1.17	1.18	3.82	3.90	
Player 9	1.83	2.14	2.01	6.77	7.08	1.34	1.36	1.17	1.10	3.70	3.87	
Player 10	1.88	2.12	2.20	6.97	7.10	1.34	1.32	1.13	1.17	3.71	3.78	

Figure S4 The calculation of Limb Symmetry Index, LSI for single-hop test distance.

Consecutive players	Single-leg hop test distance right leg [m]	Triple hop test distance left leg [m]	Triple hop test distance right leg [m]	Six-meter timed hop test time left leg [s]	Six-meter timed hop test time right leg [s]	Normalized single-leg hop test distance left leg [m*m^{-1}]	Normalized single-leg hop test distance right leg [m*m^{-1}]	Normalized triple hop test distance left leg [m*m^{-1}]	Normalized triple hop test distance right leg [m*m^{-1}]	Single-leg hop test distance LSI	Triple hop test distance LSI	Six-meter timed hop test time LSI
Player 1	2.30	7.65	7.98	1.34	1.28	1.27	1.26	4.18	4.36	98.71	95.86	95.75
Player 2	2.32	6.95	7.36	1.31	1.29	1.26	1.28	3.84	4.07	98.28	94.43	98.32
Player 3	2.13	6.88	6.85	1.34	1.32	1.17	1.18	3.82	3.81	99.06	99.56	98.14
Player 4	2.06	6.49	7.05	1.35	1.39	1.05	1.08	3.40	3.69	97.57	92.06	97.48
Player 5	1.95	6.63	6.51	1.44	1.43	1.08	1.06	3.60	3.54	97.99	98.19	98.89
Player 6	2.10	6.69	6.65	1.40	1.46	1.16	1.15	3.68	3.65	99.53	99.40	96.29
Player 7	1.83	6.82	6.16	1.48	1.61	1.10	0.99	3.69	3.33	89.71	90.32	91.51
Player 8	2.13	6.88	7.02	1.45	1.36	1.17	1.18	3.82	3.90	98.59	98.01	93.80
Player 9	2.01	6.77	7.08	1.34	1.36	1.17	1.10	3.70	3.87	93.93	95.62	98.39
Player 10	2.20	6.97	7.10	1.34	1.32	1.13	1.17	3.71	3.78	96.36	98.17	98.14

Figure S5 Calculated Limb Symmetry Index, LSI for single-leg hop test distance, triple hop test distance and six-meter timed hop test time.

4. Using the formula presented in Figure S6, calculate the arithmetic mean of six-meter timed hop test time separately for left and right legs (s), normalized single-leg hop test distance separately for left and right legs (m*m^{-1}), normalized triple hop test distance separately for left and right legs (m*m^{-1}), single-leg hop test distance LSI, triple hop test distance LSI, six-meter timed hop test time LSI in a given team as presented in Figure S7.

G12 | =AVERAGE(G2:G11)

	A	F	G	H	I	J	K	L	M	N	O	P
1	Consecutive players	Triple hop test distance right leg [m]	Six-meter timed hop test time left leg [s]	Six-meter timed hop test time right leg [s]	Normalized single-leg hop test distance left leg [m*m^{-1}]	Normalized single-leg hop test distance right leg [m*m^{-1}]	Normalized triple hop test distance left leg [m*m^{-1}]	Normalized triple hop test distance right leg [m*m^{-1}]	Single-leg hop test distance LSI	Triple hop test distance LSI	Six-meter timed hop test time LSI	
2	Player 1	7.98	1.34	1.28	1.27	1.26	4.18	4.36	98.71	95.86	95.75	
3	Player 2	7.36	1.31	1.29	1.26	1.28	3.84	4.07	98.28	94.43	98.32	
4	Player 3	6.85	1.34	1.32	1.17	1.18	3.82	3.81	99.06	99.56	98.14	
5	Player 4	7.05	1.35	1.39	1.05	1.08	3.40	3.69	97.57	92.06	97.48	
6	Player 5	6.51	1.44	1.43	1.08	1.06	3.60	3.54	97.99	98.19	98.89	
7	Player 6	6.65	1.40	1.46	1.16	1.15	3.68	3.65	99.53	99.40	96.29	
8	Player 7	6.16	1.48	1.61	1.10	0.99	3.69	3.33	89.71	90.32	91.51	
9	Player 8	7.02	1.45	1.36	1.17	1.18	3.82	3.90	98.59	98.01	93.80	
10	Player 9	7.08	1.34	1.36	1.17	1.10	3.70	3.87	93.93	95.62	98.39	
11	Player 10	7.10	1.34	1.32	1.13	1.17	3.71	3.78	96.36	98.17	98.14	
12		**MEAN**	=AVERAGE(G2:G11)									
13												

Figure S6 The formula for calculating the arithmetic mean in a given team for the left leg's six-meter timed hop test time.

A14

	A	F	G	H	I	J	K	L	M	N	O	P
1	Consecutive players	Triple hop test distance right leg [m]	Six-meter timed hop test time left leg [s]	Six-meter timed hop test time right leg [s]	Normalized single-leg hop test distance left leg [m*m^{-1}]	Normalized single-leg hop test distance right leg [m*m^{-1}]	Normalized triple hop test distance left leg [m*m^{-1}]	Normalized triple hop test distance right leg [m*m^{-1}]	Single-leg hop test distance LSI	Triple hop test distance LSI	Six-meter timed hop test time LSI	
2	Player 1	7.98	1.34	1.28	1.27	1.26	4.18	4.36	98.71	95.86	95.75	
3	Player 2	7.36	1.31	1.29	1.26	1.28	3.84	4.07	98.28	94.43	98.32	
4	Player 3	6.85	1.34	1.32	1.17	1.18	3.82	3.81	99.06	99.56	98.14	
5	Player 4	7.05	1.35	1.39	1.05	1.08	3.40	3.69	97.57	92.06	97.48	
6	Player 5	6.51	1.44	1.43	1.08	1.06	3.60	3.54	97.99	98.19	98.89	
7	Player 6	6.65	1.40	1.46	1.16	1.15	3.68	3.65	99.53	99.40	96.29	
8	Player 7	6.16	1.48	1.61	1.10	0.99	3.69	3.33	89.71	90.32	91.51	
9	Player 8	7.02	1.45	1.36	1.17	1.18	3.82	3.90	98.59	98.01	93.80	
10	Player 9	7.08	1.34	1.36	1.17	1.10	3.70	3.87	93.93	95.62	98.39	
11	Player 10	7.10	1.34	1.32	1.13	1.17	3.71	3.78	96.36	98.17	98.14	
12		**MEAN**	**1.38**	**1.38**	**1.16**	**1.15**	**3.74**	**3.80**	**96.97**	**96.16**	**96.67**	
13												

Figure S7 The calculated arithmetic mean of six-meter timed hop test time separately for left and right legs (s), normalized single-leg hop test distance separately for left and right legs (m*m-1), normalized triple hop test distance separately for left and right legs (m*m-1), single-leg hop test distance Limb Symmetry Index, LSI, triple hop test distance LSI, and six-meter timed hop test time LSI in a given team.

5. Using the formula presented in Figure S8, calculate the standard deviation of athletes of six-meter timed hop test time separately for left and right legs (s), normalized single-leg hop test distance separately for left and right legs (m*m^{-1}), normalized triple hop test distance separately for left and right legs (m*m^{-1}), single-leg hop test distance LSI, triple hop test distance LSI, six-meter timed hop test time LSI in a given team as presented in Figure S9.

G2 | =STDEV(G2:G11)

	A	F	G	H	I	J	K	L	M	N	O	P
1	Consecutive players	Triple hop test distance right leg [m]	Six-meter timed hop test time left leg [s]	Six-meter timed hop test time right leg [s]	Normalized single-leg hop test distance left leg [m*m^{-1}]	Normalized single-leg hop test distance right leg [m*m^{-1}]	Normalized triple hop test distance left leg [m*m^{-1}]	Normalized triple hop test distance right leg [m*m^{-1}]	Single-leg hop test distance LSI	Triple hop test distance LSI	Six-meter timed hop test time LSI	
2	Player 1	7.98	1.34	1.28	1.27	1.26	4.18	4.36	98.71	95.86	95.75	
3	Player 2	7.36	1.31	1.29	1.26	1.28	3.84	4.07	98.28	94.43	98.32	
4	Player 3	6.85	1.34	1.32	1.17	1.18	3.82	3.81	99.06	99.56	98.14	
5	Player 4	7.05	1.35	1.39	1.05	1.08	3.40	3.69	97.57	92.06	97.48	
6	Player 5	6.51	1.44	1.43	1.08	1.06	3.60	3.54	97.99	98.19	98.89	
7	Player 6	6.65	1.40	1.46	1.16	1.15	3.68	3.65	99.53	99.40	96.29	
8	Player 7	6.16	1.48	1.61	1.10	0.99	3.69	3.33	89.71	90.32	91.51	
9	Player 8	7.02	1.45	1.36	1.17	1.18	3.82	3.90	98.59	98.01	93.80	
10	Player 9	7.08	1.34	1.36	1.17	1.10	3.70	3.87	93.93	95.62	98.39	
11	Player 10	7.10	1.34	1.32	1.13	1.17	3.71	3.78	96.36	98.17	98.14	
12		**MEAN**	**1.38**	**1.38**	**1.16**	**1.15**	**3.74**	**3.80**	**96.97**	**96.16**	**96.67**	
13			**=STDEV(G2:G11)**									
14												

Figure S8 The formula for calculating the standard deviation for a given team's left leg's six-meter timed hop test time.

Q16 | fx

	A	F	G	H	I	J	K	L	M	N	O	P
1	Consecutive players	Triple hop test distance right leg [m]	Six-meter timed hop test time left leg [s]	Six-meter timed hop test time right leg [s]	Normalized single-leg hop test distance left leg [m*m^{-1}]	Normalized single-leg hop test distance right leg [m*m^{-1}]	Normalized triple hop test distance left leg [m*m^{-1}]	Normalized triple hop test distance right leg [m*m^{-1}]	Single-leg hop test distance LSI	Triple hop test distance LSI	Six-meter timed hop test time LSI	
2	Player 1	7.98	1.34	1.28	1.27	1.26	4.18	4.36	98.71	95.86	95.75	
3	Player 2	7.36	1.31	1.29	1.26	1.28	3.84	4.07	98.28	94.43	98.32	
4	Player 3	6.85	1.34	1.32	1.17	1.18	3.82	3.81	99.06	99.56	98.14	
5	Player 4	7.05	1.35	1.39	1.05	1.08	3.40	3.69	97.57	92.06	97.48	
6	Player 5	6.51	1.44	1.43	1.08	1.06	3.60	3.54	97.99	98.19	98.89	
7	Player 6	6.65	1.40	1.46	1.16	1.15	3.68	3.65	99.53	99.40	96.29	
8	Player 7	6.16	1.48	1.61	1.10	0.99	3.69	3.33	89.71	90.32	91.51	
9	Player 8	7.02	1.45	1.36	1.17	1.18	3.82	3.90	98.59	98.01	93.80	
10	Player 9	7.08	1.34	1.36	1.17	1.10	3.70	3.87	93.93	95.62	98.39	
11	Player 10	7.10	1.34	1.32	1.13	1.17	3.71	3.78	96.36	98.17	98.14	
12		**MEAN**	**1.38**	**1.38**	**1.16**	**1.15**	**3.74**	**3.80**	**96.97**	**96.16**	**96.67**	
13		**STDEV**	**0.06**	**0.10**	**0.07**	**0.09**	**0.20**	**0.28**	**3.02**	**3.13**	**2.39**	
14												

Figure S9 The calculated standard deviation of six-meter timed hop test time separately for left and right legs (s), normalized single-leg hop test distance separately for left and right legs (m*m-1), normalized triple hop test distance separately for left and right legs (m*m-1), single-leg hop test distance Limb Symmetry Index, LSI, triple hop test distance LSI, and six-meter timed hop test time LSI in a given team.

6. Using the formulas presented in Figure S10 and Figure S11, calculate each athlete's z-score for studied variables. The formula for calculating a z-score is z = (x-μ)/σ, where x is the score of the particular athlete, μ is the mean in a given team, and σ is the standard deviation in a given team. In the six-meter timed hop test time, conversely to the remaining tests, a higher value indicates worse performance; the z-score has to be multiplied by negative 1 (Figure S10). The z-scores have to be calculated for the six-meter timed hop test time separately for left and right legs, normalized single-leg hop test distance separately for left and right legs, normalized triple hop test distance separately for left and right legs, single-leg hop test distance LSI, triple hop test distance LSI, and six-meter timed hop test time LSI as presented in Figure S12.

P2 | fx =((G2-$G$12)/$G$13)*-1

	A	F	G	H	I	J	K	L	M	N	O	P	Q
1	Consecutive players	Triple hop test distance right leg [m]	Six-meter timed hop test time left leg [s]	Six-meter timed hop test time right leg [s]	Normalized single-leg hop test distance left leg [m*m^{-1}]	Normalized single-leg hop test distance right leg [m*m^{-1}]	Normalized triple hop test distance left leg [m*m^{-1}]	Normalized triple hop test distance right leg [m*m^{-1}]	Single-leg hop test distance LSI	Triple hop test distance LSI	Six-meter timed hop test time LSI	Six-meter timed hop test time left leg **z-score**	Six-meter timed hop test time right leg **z-score**
2	Player 1	7.98	1.34	1.28	1.27	1.26	4.18	4.36	98.71	95.86	95.75	=((G2-$G$12)/$G$13)*-1	
3	Player 2	7.36	1.31	1.29	1.26	1.28	3.84	4.07	98.28	94.43	98.32		
4	Player 3	6.85	1.34	1.32	1.17	1.18	3.82	3.81	99.06	99.56	98.14		
5	Player 4	7.05	1.35	1.39	1.05	1.08	3.40	3.69	97.57	92.06	97.48		
6	Player 5	6.51	1.44	1.43	1.08	1.06	3.60	3.54	97.99	98.19	98.89		
7	Player 6	6.65	1.40	1.46	1.16	1.15	3.68	3.65	99.53	99.40	96.29		
8	Player 7	6.16	1.48	1.61	1.10	0.99	3.69	3.33	89.71	90.32	91.51		
9	Player 8	7.02	1.45	1.36	1.17	1.18	3.82	3.90	98.59	98.01	93.80		
10	Player 9	7.08	1.34	1.36	1.17	1.10	3.70	3.87	93.93	95.62	98.39		
11	Player 10	7.10	1.34	1.32	1.13	1.17	3.71	3.78	96.36	98.17	98.14		
12		**MEAN**	**1.38**	**1.38**	**1.16**	**1.15**	**3.74**	**3.80**	**96.97**	**96.16**	**96.67**		
13		**STDEV**	**0.06**	**0.10**	**0.07**	**0.09**	**0.20**	**0.28**	**3.02**	**3.13**	**2.39**		

Figure S10 The formula for calculating the z-score for a left leg's six-meter timed hop test time.

R2 | =(I2-$I$12)/$I$13

	A	H	I	J	K	L	M	N	O	P	Q	R	S
1	Consecutive players	Six-meter timed hop test time right leg [s]	Normalized single-leg hop test distance left leg [m*m-1]	Normalized single-leg hop test distance right leg [m*m-1]	Normalized triple hop test distance left leg [m*m-1]	Normalized triple hop test distance right leg [m*m-1]	Single-leg hop test distance LSI	Triple hop test distance LSI	Six-meter timed hop test time LSI	Six-meter timed hop test time left leg z-score	Six-meter timed hop test time right leg z-score	Normalized single-leg hop test distance left leg z-score	Normalized single-leg hop test distance right leg z-score
2	Player 1	1.28	1.27	1.26	4.18	4.36	98.71	95.86	95.75	0.69		=(I2-$I$12)/$I$13	
3	Player 2	1.29	1.26	1.28	3.84	4.07	98.28	94.43	98.32	1.18			
4	Player 3	1.32	1.17	1.18	3.82	3.81	99.06	99.56	98.14	0.68			
5	Player 4	1.39	1.05	1.08	3.40	3.69	97.57	92.06	97.48	0.47			
6	Player 5	1.43	1.08	1.06	3.60	3.54	97.99	98.19	98.89	-1.06			
7	Player 6	1.46	1.16	1.15	3.68	3.65	99.53	99.40	96.29	-0.36			
8	Player 7	1.61	1.10	0.99	3.69	3.33	89.71	90.32	91.51	-1.66			
9	Player 8	1.36	1.17	1.18	3.82	3.90	98.59	98.01	93.80	-1.25			
10	Player 9	1.36	1.17	1.10	3.70	3.87	93.93	95.62	98.39	0.68			
11	Player 10	1.32	1.13	1.17	3.71	3.78	96.36	98.17	98.14	0.64			
12		1.38	1.16	1.15	3.74	3.80	96.97	96.16	96.67				
13		0.10	0.07	0.09	0.20	0.28	3.02	3.13	2.39				

Figure S11 The formula for calculating the z-score for a left leg's normalized single-leg hop test distance.

A14

	A	F	G	H	I	J	K	L	M	N	O	P	Q	R	S	T	U	V	W	X
1	Consecutive players	Triple hop test distance right leg [m]	Six-meter timed hop test time left leg [s]	Six-meter timed hop test time right leg [s]	Normalized single-leg hop test distance left leg [m*m-1]	Normalized single-leg hop test distance right leg [m*m-1]	Normalized triple hop test distance left leg [m*m-1]	Normalized triple hop test distance right leg [m*m-1]	Single-leg hop test distance LSI	Triple hop test distance LSI	Six-meter timed hop test time LSI	Six-meter timed hop test time left leg z-score	Six-meter timed hop test time right leg z-score	Normalized single-leg hop test distance left leg z-score	Normalized single-leg hop test distance right leg z-score	Normalized triple hop test distance left leg z-score	Normalized triple hop test distance right leg z-score	Single-leg hop test distance LSI z-score	Triple hop test distance LSI z-score	Six-meter timed hop test time LSI z-score
2	Player 1	7.98	1.34	1.28	1.27	1.26	4.18	4.36	98.71	95.86	95.75	0.69	0.99	1.65	1.24	2.18	1.98	0.58	-0.10	-0.39
3	Player 2	7.36	1.31	1.29	1.26	1.28	3.84	4.07	98.28	94.43	98.32	1.18	0.92	1.46	1.51	0.48	0.94	0.43	-0.55	0.69
4	Player 3	6.85	1.34	1.32	1.17	1.18	3.82	3.81	99.06	99.56	98.14	0.68	0.66	0.22	0.42	0.39	0.02	0.69	1.09	0.61
5	Player 4	7.05	1.35	1.39	1.05	1.08	3.40	3.69	97.57	92.06	97.48	0.47	-0.07	-1.47	-0.74	-1.72	-0.38	0.20	-1.31	0.34
6	Player 5	6.51	1.44	1.43	1.08	1.06	3.60	3.54	97.99	98.19	98.89	-1.06	-0.44	-1.06	-0.95	-0.70	-0.92	0.34	0.65	0.93
7	Player 6	6.65	1.40	1.46	1.16	1.15	3.68	3.65	99.53	99.40	96.29	-0.36	-0.75	0.04	0.09	-0.34	-0.51	0.85	1.04	-0.16
8	Player 7	6.16	1.48	1.61	1.10	0.99	3.69	3.33	89.71	90.32	91.51	-1.66	-2.34	-0.76	-1.74	-0.28	-1.66	-2.41	-1.87	-2.16
9	Player 8	7.02	1.45	1.36	1.17	1.18	3.82	3.90	98.59	98.01	93.80	-1.25	0.20	0.14	0.42	0.39	0.36	0.54	0.59	-1.20
10	Player 9	7.08	1.34	1.36	1.17	1.10	3.70	3.87	93.93	95.62	98.39	0.68	0.19	0.18	-0.52	-0.22	0.25	-1.01	-0.17	0.72
11	Player 10	7.10	1.34	1.32	1.13	1.17	3.71	3.78	96.36	98.17	98.14	0.64	0.64	-0.41	0.27	-0.18	-0.08	-0.20	0.64	0.61
12		MEAN	1.38	1.38	1.16	1.15	3.74	3.80	96.97	96.16	96.67									
13		STDEV	0.06	0.10	0.07	0.09	0.20	0.28	3.02	3.13	2.39									
14																				

Figure S12 Calculated z-scores for the six-meter timed hop test time separately for left and right legs, normalized single-leg hop test distance separately for left and right legs, normalized triple hop test distance separately for left and right legs, single-leg hop test distance LSI, triple hop test distance LSI, and six-meter timed hop test time LSI.

7. Using the formula presented in Figure S13, calculate the Compound Hop Index, CHI for each athlete by averaging obtained z-scores for the six-meter timed hop test time separately for left and right legs, normalized single-leg hop test distance separately for left and right legs, normalized triple hop test distance separately for left and right legs, single-leg hop test distance LSI, triple hop test distance LSI, and six-meter timed hop test time LSI (Figure S14).

P2 | =AVERAGE(P2:X2)

	A	G	H	I	J	K	L	M	N	O	P	Q	R	S	T	U	V	W	X	Y	Z
1	Consecutive players	Six-meter timed hop test time left leg [s]	Six-meter timed hop test time right leg [s]	Normalized single-leg hop test distance left leg [m*m-1]	Normalized single-leg hop test distance right leg [m*m-1]	Normalized triple hop test distance left leg [m*m-1]	Normalized triple hop test distance right leg [m*m-1]	Single-leg hop test distance LSI	Triple hop test distance LSI	Six-meter timed hop test time LSI	Six-meter timed hop test time left leg z-score	Six-meter timed hop test time right leg z-score	Normalized single-leg hop test distance left leg z-score	Normalized single-leg hop test distance right leg z-score	Normalized triple hop test distance left leg z-score	Normalized triple hop test distance right leg z-score	Single-leg hop test distance LSI z-score	Triple hop test distance LSI z-score	Six-meter timed hop test time LSI z-score	CHI	
2	Player 1	1.34	1.28	1.27	1.26	4.18	4.36	98.71	95.86	95.75	0.69	0.99	1.65	1.24	2.18	1.98	0.58	-0.10	-0.39	=AVERAGE(P2:X2)	
3	Player 2	1.31	1.29	1.26	1.28	3.84	4.07	98.28	94.43	98.32	1.18	0.92	1.46	1.51	0.48	0.94	0.43	-0.55	0.69		
4	Player 3	1.34	1.32	1.17	1.18	3.82	3.81	99.06	99.56	98.14	0.68	0.66	0.22	0.42	0.39	0.02	0.69	1.09	0.61		
5	Player 4	1.35	1.39	1.05	1.08	3.40	3.69	97.57	92.06	97.48	0.47	-0.07	-1.47	-0.74	-1.72	-0.38	0.20	-1.31	0.34		
6	Player 5	1.44	1.43	1.08	1.06	3.60	3.54	97.99	98.19	98.89	-1.06	-0.44	-1.06	-0.95	-0.70	-0.92	0.34	0.65	0.93		
7	Player 6	1.40	1.46	1.16	1.15	3.68	3.65	99.53	99.40	96.29	-0.36	-0.75	0.04	0.09	-0.34	-0.51	0.85	1.04	-0.16		
8	Player 7	1.48	1.61	1.10	0.99	3.69	3.33	89.71	90.32	91.51	-1.66	-2.34	-0.76	-1.74	-0.28	-1.66	-2.41	-1.87	-2.16		
9	Player 8	1.45	1.36	1.17	1.18	3.82	3.90	98.59	98.01	93.80	-1.25	0.20	0.14	0.42	0.39	0.36	0.54	0.59	-1.20		
10	Player 9	1.34	1.36	1.17	1.10	3.70	3.87	93.93	95.62	98.39	0.68	0.19	0.18	-0.52	-0.22	0.25	-1.01	-0.17	0.72		
11	Player 10	1.34	1.32	1.13	1.17	3.71	3.78	96.36	98.17	98.14	0.64	0.64	-0.41	0.27	-0.18	-0.08	-0.20	0.64	0.61		
12		1.38	1.38	1.16	1.15	3.74	3.80	96.97	96.16	96.67											
13		0.06	0.10	0.07	0.09	0.20	0.28	3.02	3.13	2.39											
14																					

Figure S13 The formula for calculating the Compound Hop Index, CHI for an athlete in a given team.

Y1 CHI

	A	B	C	D	E	F	G	H	I	J	K	L
1	Consecutive players	Body height [m]	Single-leg hop test distance left leg [m]	Single-leg hop test distance right leg [m]	Triple hop test distance left leg [m]	Triple hop test distance right leg [m]	Six-meter timed hop test time left leg [s]	Six-meter timed hop test time right leg [s]	Normalized single-leg hop test distance left leg [m*m^{-1}]	Normalized single-leg hop test distance right leg [m*m^{-1}]	Normalized triple hop test distance left leg [m*m^{-1}]	Normalized triple hop test distance right leg [m*m^{-1}]
2	Player 1	1.83	2.33	2.30	7.65	7.98	1.34	1.28	1.27	1.26	4.18	4.36
3	Player 2	1.81	2.28	2.32	6.95	7.36	1.31	1.29	1.26	1.28	3.84	4.07
4	Player 3	1.80	2.11	2.13	6.88	6.85	1.34	1.32	1.17	1.18	3.82	3.81
5	Player 4	1.91	2.01	2.06	6.49	7.05	1.35	1.39	1.05	1.08	3.40	3.69
6	Player 5	1.84	1.99	1.95	6.63	6.51	1.44	1.43	1.08	1.06	3.60	3.54
7	Player 6	1.82	2.11	2.10	6.69	6.65	1.40	1.46	1.16	1.15	3.68	3.65
8	Player 7	1.85	2.04	1.83	6.82	6.16	1.48	1.61	1.10	0.99	3.69	3.33
9	Player 8	1.80	2.10	2.13	6.88	7.02	1.45	1.36	1.17	1.18	3.82	3.90
10	Player 9	1.83	2.14	2.01	6.77	7.08	1.34	1.36	1.17	1.10	3.70	3.87
11	Player 10	1.88	2.12	2.20	6.97	7.10	1.34	1.32	1.13	1.17	3.71	3.78
12						**MEAN**	**1.38**	**1.38**	**1.16**	**1.15**	**3.74**	**3.80**
13						**STDEV**	**0.06**	**0.10**	**0.07**	**0.09**	**0.20**	**0.28**

	A	M	N	O	P	Q	R	S	T	U	V	W	X	Y
1	Consecutive players	Single-leg hop test distance LSI	Triple hop test distance LSI	Six-meter timed hop test time LSI	Six-meter timed hop test time left leg **z-score**	Six-meter timed hop test time right leg **z-score**	Normalized single-leg hop test distance left leg **z-score**	Normalized single-leg hop test distance right leg **z-score**	Normalized triple hop test distance left leg **z-score**	Normalized triple hop test distance right leg **z-score**	Single-leg hop test distance LSI **z-score**	Triple hop test distance LSI **z-score**	Six-meter timed hop test time LSI **z-score**	CHI
2	Player 1	98.71	95.86	95.75	0.69	0.99	1.65	1.24	2.18	1.98	0.58	-0.10	-0.39	0.98
3	Player 2	98.28	94.43	98.32	1.18	0.92	1.46	1.51	0.48	0.94	0.43	-0.55	0.69	0.79
4	Player 3	99.06	99.56	98.14	0.68	0.66	0.22	0.42	0.39	0.02	0.69	1.09	0.61	0.53
5	Player 4	97.57	92.06	97.48	0.47	-0.07	-1.47	-0.74	-1.72	-0.38	0.20	-1.31	0.34	-0.52
6	Player 5	97.99	98.19	98.89	-1.06	-0.44	-1.06	-0.95	-0.70	-0.92	0.34	0.65	0.93	-0.36
7	Player 6	99.53	99.40	96.29	-0.36	-0.75	0.04	0.09	-0.34	-0.51	0.85	1.04	-0.16	-0.01
8	Player 7	89.71	90.32	91.51	-1.66	-2.34	-0.76	-1.74	-0.28	-1.66	-2.41	-1.87	-2.16	-1.65
9	Player 8	98.59	98.01	93.80	-1.25	0.20	0.14	0.42	0.39	0.36	0.54	0.59	-1.20	0.02
10	Player 9	93.93	95.62	98.39	0.68	0.19	0.18	-0.52	-0.22	0.25	-1.01	-0.17	0.72	0.01
11	Player 10	96.36	98.17	98.14	0.64	0.64	-0.41	0.27	-0.18	-0.08	-0.20	0.64	0.61	0.22
12	**MEAN**	**96.97**	**96.16**	**96.67**										
13	**STDEV**	**3.02**	**3.13**	**2.39**										

Figure S14 Compound Hop Index, CHI calculated for each athlete in a given team.
