# Supplementary material for: A Compound Hop Index for Assessing Soccer Players’ Performance
Source: J Clin Med. 2022 Jan 4;11(1):255. doi: 10.3390/jcm11010255 (PMC8745790; doi:10.3390/jcm11010255)
Supplement: Supplementary file 1 [file jcm-11-00255-s001.zip › jcm-1495912-supplementary/S2_Table_List of calculated CHI for particular players of a given team.pdf]

Table S1. List of calculated CHI for particular players of a given team.

Consecutive players	CHI
Player 1	0.85
Player 2	0.53
Player 3	0.35
Player 4	−0.63
Player 5	−0.29
Player 6	0.09
Player 7	−1.32
Player 8	0.19
Player 9	−0.17
Player 10	0.03
Player 11	0.37
Player 12	0.32
Player 13	0.60
Player 14	−2.50
Player 15	0.54
Player 16	0.27
Player 17	−1.43
Player 18	0.02
Player 19	−0.16
Player 20	−0.76
Player 21	0.29
Player 22	0.68
Player 23	−0.49
Player 24	−0.37
Player 25	0.14
Player 26	−0.32
Player 27	−0.07
Player 28	0.42
Player 29	0.34
Player 30	0.60
Player 31	0.69
Player 32	0.77
Player 33	−0.96
Player 34	0.06
Player 35	−0.11
Player 36	1.20
Player 37	0.30
Player 38	−0.58
Player 39	−0.03
Player 40	0.05
Player 41	0.62
Player 42	−0.45
Player 43	0.31
